# The history and mystery of sacubitril/valsartan: From clinical trial to the real world

**DOI:** 10.3389/fcvm.2023.1102521

**Published:** 2023-03-28

**Authors:** Mingsong Zhang, Yifei Zou, Yangxue Li, He Wang, Wei Sun, Bin Liu

**Affiliations:** Department of Cardiology, The Second Hospital of Jilin University, Changchun, China

**Keywords:** heart failure, sacubitril/valsartan, cardiovascular, clinical application, basic medical research

## Abstract

Heart failure is a serious threat to human health, with morbidity and mortality rates increasing despite the existence of multiple treatment options. Therefore, it is necessary to identify new therapeutic targets for this disease. Sacubitril/valsartan is a supramolecular sodium salt complex of the enkephalinase inhibitor prodrug sacubitril and the angiotensin receptor blocker valsartan. Its combined action increases endogenous natriuretic peptides while inhibiting the renin-angiotensin-aldosterone system and exerting cardioprotective effects. Clinical evidence suggests that sacubitril/valsartan is superior to conventional renin-angiotensin-aldosterone inhibitor therapy for patients with reduced ejection fraction heart failure who can tolerate angiotensin-converting enzyme inhibitors or angiotensin II receptor blockers. The therapy reduces the risk of heart failure hospitalization, cardiovascular mortality, and all-cause mortality and has a better safety and tolerability record. This review describes the potential pathophysiological mechanisms of cardiomyocyte injury amelioration by sacubitril/valsartan. We explore the protective effects of sacubitril/valsartan and outline the therapeutic value in patients with heart failure by summarizing the results of recent large clinical trials. Furthermore, a preliminary outlook shows that sacubitril/valsartan may be effective at treating other diseases, and provides some exploratory observations that lay the foundation for future studies on this drug.

## 1. Introduction

Heart failure (HF) is a common clinical condition characterized by typical clinical symptoms such as dyspnea, edema, and decreased exercise tolerance, together with signs resulting from structural or functional abnormalities of the heart. It can be divided into three categories based on the left ventricular ejection fraction (LVEF): preserved ejection fraction (LVEF ≥ 50%), mildly reduced ejection fraction (LVEF 40%–49%), and reduced ejection fraction (LVEF < 40%). The etiology, comorbidities, and treatment of different types of HF differ; however, their pathophysiological features have similarities (1). Activation of the sympathetic nervous system is the fastest adaptive response mechanism in HF leading to positive inotropic and chronotropic effects that maintain perfusion of vital organs through blood redistribution (2). The renin-angiotensin-aldosterone (RAAS) system is also activated to maintain hemodynamic stability. However, over-activated sympathetic nerves can have negative effects, such as direct toxicity of epinephrine to cardiomyocytes, and inducing cardiomyocyte hypertrophy and apoptosis. Activation of the RAAS system leads to sodium and water retention, myocardial hypertrophy, and fibrosis (3). In addition, the reduced effectiveness of the natriuretic peptide (NP) system in HF patients can further aggravate sodium retention, vasoconstriction, and volume overload which can seriously affect long-term prognosis (4). Clinical trials showed that neither exogenous NPs administration nor enkephalinase inhibition (NEPI) alone has a good therapeutic effect, which may be a detrimental for the over-activated RAAS system (5–7). Therefore, the development and the use of drugs that effectively inhibit the activation of the neuroendocrine system in patients with HF have shown to be effective in reducing mortality and hospitalizations in HF patients so much so that they are strongly recommended by international guidelines for the treatment of heart failure (8).

**Figure 1:**
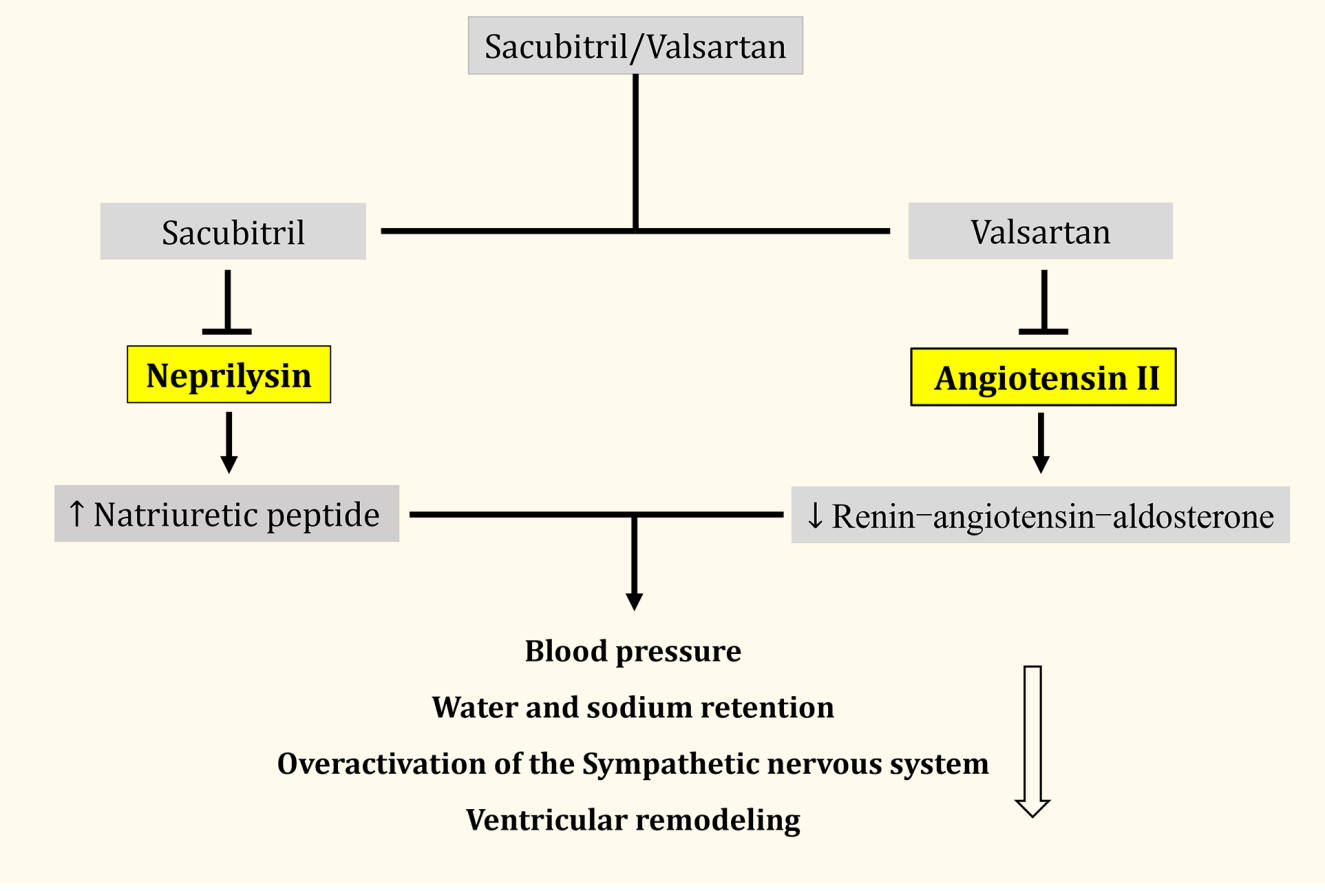
Simplified overview of the effects of sacubitril/valsartan.

Sacubitril/valsartan (Entresto, development code LCZ696) was approved by the U.S. Food and Drug Administration (FDA) and the European Medicines Agency (EMA) in 2015 as the first successful angiotensin receptor- enkephalinase inhibitor (ARNI) on the market. Sacubitril/valsartan is a 1:1 combination of the NEPI prodrug sacubitril and the angiotensin II receptor blocker (ARB), valsartan (9, 10). The metabolized active form of sacubitril (LBQ657) inhibits enkephalinase. Enkephalinase is a naturally occurring zinc-dependent membrane metallopeptidase that metabolizes different vasoactive peptides including NPs, bradykinin, and angiotensin II (Ang II) (11). Enkephalinase inhibition leads to an increase in circulating NPs, especially atrial natriuretic peptide (ANP), brain natriuretic peptide (BNP), and other vasoactive peptides. It also has a therapeutic effect on many cardiovascular diseases (CVDs) through its antioxidant-, anti-inflammatory-, and antifibrotic effects. It should be noted that LBQ657 inhibits Ang II degradation by enkephalinase which results in increased Ang II levels and accelerated progression of HF; however, its combination with valsartan resolves this problem (Figure 1) (6, 12–14). Clinical evidence suggests that sacubitril/valsartan further reduces the risk of HF hospitalization, cardiovascular mortality, and all other causes of mortality in patients with HF who can tolerate angiotensin-converting enzyme inhibitors (ACEI) or ARBs, but also has a better safety and tolerability profile (15). This review provides a comprehensive and systematic overview of the pathophysiological mechanisms of sacubitril/valsartan in CVD, and an in-depth discussion of its role in several large clinical trials. This may be used as a guide to the widespread use of sacubitril/valsartan in clinical practice.

## 2. Mechanisms of action of sacubitril valsartan: experimental evidences

### 2.1. Oxidative stress

Sacubitril/valsartan inhibits superoxide dismutase (SOD), catalase (CAT), glutathione peroxidase (GPx), glutathione S-transferase (GST), degradation of antioxidant enzymes, and oxidative stress in the isoprenaline (ISO)-induced myocardial infarction (MI) rat model (16). Furthermore, it reduces myogenic fiber damage and infarct size, and provides cardioprotective effects for the heart after MI.

Sacubitril/valsartan ameliorates cardiorenal syndrome (CRS)-mediated decreases in the expression levels of antioxidant enzymes, including NADPH oxidase 4 (NOX-4), xanthine oxidase, and CAT. It downregulates intracellular reactive oxygen species (ROS) levels, and ameliorates CRS-induced heart and kidney damage in rats (17). Furthermore, it significantly reduces the levels of nuclear transcription factor κB (NF-κB) p65, increases nuclear factor E2-related factor 2 (Nrf2) nuclear translocation levels, and improves chronic kidney disease (CKD)-mediated oxidative stress compared to valsartan alone (Table 1) (18).

**Table 1:** Mechanisms of action of sacubitril valsartan: experimental evidences.

Pathophysiological mechanisms	Sample	Models/Method	Pathway/Target	Ref.
Oxidative stress	Rats	MI induced by ISO	Degradation of SOD, CAT, GPx and GST↓	(16)
	Rats	CRS induced by 5/6 nephrectomy and intra-peritoneal doxorubicin administration together	NOX-4, xanthine-oxidase, and CAT↑→ROS↓	(17)
	Rats	CKD rats underwent 5/6 nephrectomy	Nuclear translocation of NF-κB p65↓→ nuclear translocation of Nrf2↑	(18)
Inflammation	Mice	ApoE^−/−^ mice	MMP-8, IL-6, and MCP-1↓	(19)
	HUVECs	LPS	TLR4/Myd88 pathway and nuclear translocation of NF-κB p65↓→IL-6, IL-1α, TNF-β, MCP-1, and CXCL1↓	(20)
	Human	HFrEF	TNF-α and IL-18↓	(21)
	Rats	MI by ligating the LAD	TAK1/JNK pathway↓→NLRP3 inflammasome↓	(22)
	Mice	Dox-induced chronic cardiomyopathy	TLR2-MyD88 complex↓→ degradation of IκB-a and nuclear translocation of NF-κB↓→*Tnf-α, Mcp-1, and Il-6*↓	(23)
Apoptosis	HUVECs	ox-LDL	TLR4/NF-κB pathway↓→ MALAT1↓	(24)
	Rats	CRS rats	Mfn2↓→mitochondrial fission↓	(25)
	Rats	DCM rats	AGEs, RAGE, CHOP, and PERK↓→ ER stress↓	(26)
	Rats/H9c2	Dox-induced cardiotoxicity	GRP78, PERK, IRE-1α, eIF-2α, ATF-4, CHOP, Bax, and Caspase-3↓→ER stress↓	(27)
	Mice	DCM induced by Dox	Drp1 and Drp1 Ser616 phosphorylation↓→mitochondrial fission↓	(28)
Cardiac hypertrophy	Mice	MI by ligating the LAD	*Anp* and *β-Mhc*↓	(29)
	Rats	TAC rats	*Myh7*↓	(30)
	Mice	TAC mice	mRNA of *Anp*, *Bnp*, and *β-Mhc*↓	(31)
	NRCMs	PE	Sirt3/MnSOD and the ratio of p-AMPK/AMPK↑	(31)
Cardiac fibrosis	Rats	MI by ligating the LAD	TGF-β1/Smads pathway↓	(32)
	Rats	MI by ligating the LAD	Circulating exosomes-miR-181a, *Fn1* and *Col1*↓	(33)
	Rats	MI by ligating the LAD	mRNA of *α-Sma* and *Tgf-β*↓, β-catenin↓, sFRP-1↑	(34)
	CFs	Ang II	sFRP-1↑→ Wnt/β-catenin pathway↓	(34)
	Mice	TAC mice	*Acta2, Col1a1*, and *Postn*↓	(35)
	Human CFs	TGF-β1/Ang II	NPR-cGMP-PKG pathway↑→RhoA↓	(35)
	MEF	TGF-β1	Function of TRPM7 channel↓	(36)

α-SMA, α-smooth muscle actin; ATF-4, Activating transcription factor 4; ANP, Atrial natriuretic peptide; AGEs, Advanced glycation end products; Ang II, Angiotensin II; β-MHC, β-Myosin heavy chain; BNP, Brain natriuretic peptide; CAT, Catalases; CXCL1, Chemokine C-X-C motif ligand-1; CHOP, C/EBP homologous protein; CRS, Cardiorenal syndrome; CKD, Chronic kidney disease; CFs, Cardiac fibroblasts; cGMP, Cyclic guanosine monophosphate; DOX, Doxorubicin; DCM, Dilated cardiomyopathy; eIF-2α, Eukaryotic translation initiation factor 2; ER, Endoplasmic reticulum; GPx, Glutathione peroxidase; GST, Glutathione S-transferase; GRP78, Glucose-Regulated Protein 78; HUVECs, Human umbilical vein endothelial cells; HFrEF, Heart failure with reduced ejection fraction;.

ISO, Isoproterenol; IL-6, Interleukin-6; IL-1α, Interleukin-1α; IRE-1α, Inositol requiring enzyme 1; LAD, Left anterior descending; LPS, Lipopolysaccharide; MI, Myocardial infarction; MMP-8, Matrix metalloproteinase-8; MCP-1, Monocyte chemoattractant protein-1; Mfn2, Mitofusin 2;NOX-4, NADPH oxidase 4;Nrf2, Nuclear factor E2-related factor 2; NPR, Natriuretic peptide receptor; ox-LDL, Oxidized low-density lipoprotein; PE, Phenylephrine; PERK, PKR-like endoplasmic reticulum kinase; PKG, Protein kinase G; ROS, Reactive oxygen species; RAGE, Receptor for advanced glycation end-products; sFRP-1, Secreted frizzled-related protein 1; SOD, Superoxide dismutase; TNF-β, Tumor necrosis factor beta; TNF-α, Tumor necrosis factor alpha; TAC, Transverse aortic constriction; TRPM7, Transient receptor potential melastatin-subfamily member 7.

### 2.2. Inflammation

Atherosclerotic plaque formation is an inflammatory condition of the arterial wall. The expression of pro-inflammatory cytokines, including matrix metalloproteinase-8 (MMP-8), interleukin-6 (IL-6), and monocyte chemoattractant protein-1 (MCP-1) is downregulated in AS plaques of apolipoprotein E-deficient mice (apoE^−/−^ mice) that were fed a high-fat diet after sacubitril/valsartan treatment. Furthermore, atherosclerotic plaque area and lipid content were reduced, collagen content and fibrous cap thickness were increased, and plaque stability was improved (19). Sacubitril/valsartan downregulates the expression of pro-inflammatory cytokines IL-6, IL-1α, tumor necrosis factor (TNF-β), chemokine (MCP-1), and chemokine CXC motif ligand-1 (CXCL1) by inhibiting the Toll-like receptor 4 (TLR4)/myeloid differentiation factor 88 (Myd88) pathway, NF-κB p65 nuclear translocation, and improving lipopolysaccharide (LPS)-induced endothelial cell inflammation (20). It significantly reduces circulating TNF-α and IL-18 levels in patients with HF with reduced ejection fraction (HFrEF) after 4 weeks of treatment, accompanied by improved peripheral vascular function and increased exercise tolerance (21).

Sacubitril/valsartan inhibits the inflammatory response and improves ventricular remodeling by blocking the TAK1/JNK signaling pathway and reducing the expression level of the NLRP3 inflammasome in rats with HF after MI (22). It inhibits formation of the TLR2-MyD88 complex, reverses DOX-induced degradation of IκB-α and increase of nuclear translocation of NF-κB, and downregulates the expression of pro-inflammatory genes, including TNF-α, MCP-1, and IL-6 in a mouse model of doxorubicin (DOX)-induced chronic cardiomyopathy; this causes a reduction in cardiac inflammation and amelioration of cardiac injury (23).

### 2.3. Apoptosis

Sacubitril/valsartan inhibits oxidized low-density lipoprotein-induced apoptosis in human umbilical vein endothelial cells and ameliorates endothelial injury by inhibiting the TLR4/NF-κB signaling pathway and downregulating metastasis-associated lung adenocarcinoma transcript 1 (MALAT1) expression (24). Administration of sacubitril/valsartan to rats fed a high-protein diet with CRS downregulates mitofusin 2 (Mfn2) expression, inhibits mitochondrial division, protects the functional integrity of mitochondria, inhibits apoptosis, and improves cardiac function (25).

Sacubitril/valsartan significantly downregulates advanced glycation end products formation and receptor for advanced glycation end-product (RAGE) expression while decreasing C/EBP homologous protein (CHOP) and phosphorylated PKR-like endoplasmic reticulum kinase (PERK) expression levels in rats with diabetic cardiomyopathy (DCM) (26). This leads to improved endoplasmic reticulum (ER) stress and DCM-induced apoptosis.

Sacubitril/valsartan downregulates the ER stress-related proteins glucose-regulated protein 78 (GRP78), PERK, inositol-requiring enzyme 1α (IRE-1α), eukaryotic translation initiation factor 2 (eIF-2α), activating transcription factor 4 (ATF4), and CHOP expression in the DOX-induced rat cardiotoxicity model and H9c2 cardiomyocyte model (27). Furthermore, it downregulates the expression of apoptotic proteins Bax and Caspase-3, reduces cardiomyocyte apoptosis and cardiac systolic dysfunction. It protects cardiac function by downregulating Drp1 protein expression and inhibiting Drp1 Ser616 phosphorylation in mouse cardiomyocytes, thereby reducing mitochondrial fission and apoptosis (28).

### 2.4. Cardiac hypertrophy

Sacubitril/valsartan downregulates the expression of cardiac hypertrophy marker genes, including *ANP* and *β-myosin heavy chain (β-MHC)*, significantly reduced cardiomyocyte hypertrophy in the non-infarct and border zones, and attenuated adverse cardiac remodeling in a mouse model of MI constructed by ligation of the left anterior descending branch (29).

Sacubitril/valsartan treatment of an HF with preserved ejection fraction (HFpEF) rat model constructed by the transverse aortic constriction (TAC) procedure (30) significantly decreases left ventricular weight, downregulates *Myh7* gene expression, and improves myocardial hypertrophy and diastolic dysfunction. Sacubitril/valsartan downregulates the mRNA levels of cardiac hypertrophy markers, including ANP, BNP, and β-MHC in a mouse model of TAC surgery-induced HF (31). This significantly reduces the cross-sectional area of cardiomyocytes. Sacubitril/valsartan upregulates sirtuin 3 (Sirt3)/manganese superoxide dismutase (MnSOD) expression and p-AMPK/AMPK ratio to inhibit phenylephrine-induced cardiomyocyte hypertrophy *in vitro*. Sirt3 knockdown abrogates the protective effect of sacubitril/valsartan on cardiomyocyte hypertrophy. This suggests that sacubitril/valsartan activates the Sirt3/MnSOD pathway to ameliorate pathological cardiac remodeling induced by pressure overload.

### 2.5. Cardiac fibrosis

In a post-MI rat model of HF, Wu et al. found that sacubitril/valsartan significantly inhibits the upregulation of transforming growth factor β1 (TGF-β1) and p-Smad3 protein expression in the rat infarcted myocardium (32). A similar model observed that sacubitril/valsartan downregulates circulating exosome miR-181a, decrease *FN1* and *COL1* gene expression levels, and decreases the fibrosis area (33). Sacubitril/valsartan reverses the increased transcript levels of *α-smooth muscle actin* (*α-SMA*) and *TGF-β* genes, suppresses *β-catenin* expression, and upregulates *secreted frizzled-related protein 1* (*sFRP-1*) expression in rats with HF after MI. Further studies in primary mouse fibroblasts revealed that sacubitril/valsartan prevents the progression of myocardial fibrosis by inhibiting the Wnt/β-catenin pathway through SFRP1 upregulation (34).

Sacubitril/valsartan inhibits the expression of genes associated with myofibroblast transformation and activation (including *Acta2*, *Col1a1*, and *Postn*) in a mouse model of left ventricular pressure overload (35), blocks myofibroblast activation and reduces pathological accumulation of cardiac fibroblasts. Further experiments with TGF-β1/Ang II-stimulated human CF confirm that sacubitril/valsartan inhibits the function of RhoA and prevents the conversion of cardiac fibroblasts to myofibroblasts by activating the natriuretic peptide receptor (NPR)-cGMP-PKG signaling pathway. Meanwhile, LBQ657 inhibits TGF-β1-induced fibroblast activation by blocking the function of the TRPM7 channel *in vitro* (36).

## 3. Clinical trials of sacubitril/valsartan

### 3.1. PARAMOUNT

PARAMOUNT (NCT00887588) is a 36-week randomized, double-blind, multicenter, parallel-group, active-controlled phase II clinical trial (Table 2) evaluating the efficacy, safety, and tolerability of sacubitril/valsartan vs. valsartan in HF. Solomon et al. showed N-terminal pro-brain natriuretic peptide (NT-proBNP) rapidly declined from week 4 to week 12 and 36 of the primary trial endpoint in the sacubitril/valsartan-treated group compared to valsartan (37). Patients in the sacubitril/valsartan group have reduced left atrial size, reversed left atrial remodeling, significantly improved New York Heart Association (NYHA) classification, and good overall tolerability at week 36 (Table 3).

**Table 2:** Overview of sacubitril/valsartan clinical trials.

Trial	Actual Enrollment	LVEF	NYHA	Type of trial	Primary endpoint	Secondary endpoint	Follow-up time
PARAMOUNT (2015)	307	≥45%	II–IV	Randomized, double-blind, multi-center, parallel group, active controlled study	Change from baseline in NT-proBNP	Change from baseline in BNP, cGMP, ECHO parameters, and others	36 weeks
PARADIGM-HF (2015)	8442	≤35%	II–IV	Multi-center, randomized, double-blind, parallel group, active-controlled study	Number of participants that had first occurrence of the composite endpoint	Number of patients—all-cause mortality, number of patients reported with adjudicated primary causes of death, and others	51 months
PARAGON-HF (2020)	4822	≥45%	II–IV	Multicenter, randomized, double-blind, parallel group, active-controlled study	Cumulative number of primary composite events of cardiovascular death and total HF hospitalizations	Change from baseline to month 8 in the clinical summary score by KCCQ and NYHA functional class, all-cause mortality, and others	57 months
TRANSITION (2021)	1002	≤40%	II–IV	Multicenter, randomized, open Label, parallel group study	Percentage of patients achieving the target dose of LCZ696 at 10 weeks post randomization	Percentage of patients achieving and maintaining either 100 mg and/or 200 mg bid LCZ696; any dose of LCZ696; permanently discontinued from treatment	10 weeks
PRIME (2018)	118	25%–50%	II–III	Multicenter, randomized, double-blind, active-controlled study	Change of EROA in functional mitral regurgitation from baseline to 12 months follow-up	Change of regurgitant volume, left ventricular end-diastolic volume, and incomplete mitral leaflet closure area from baseline to 12 months follow-up	12 months
PIONEER-HF (2019)	887	≤40%	IV	Multicenter, randomized, double-blind, double dummy, parallel group, active-controlled study	NT-proBNP values and time-averaged change from baseline	Number of patients with incidences of symptomatic hypotension, hyperkalemia, and angioedema; change from baseline in hs-Troponin, urinary cGMP, and other changes	8 weeks
EVALUATE-HF (2020)	465	≤40%	I–III	Multicenter, randomized, double-blind, double-dummy, parallel group, active-controlled, forced-titration study	Change from baseline in aortic characteristic impedance at week 12	Pearson's correlation coefficient between change from baseline in aortic characteristic impedance and biomarker levels; change from baseline in NT-proBNP and echocardiographic measure, and other changes	12 weeks
PROVE-HF (2019)	794	≤40%	II–IV	Multicenter, open-label, single-arm study	Change in concentration of NT-proBNP, LAVi, LVEDVi, LVESVi, and LVEF from baseline to one year; change in log-transformed NT-proBNP and change in structural cardiac measurements LVESVi, LVEDVi, LAVi, and LVEF from baseline to one year	Change in log-transformed NT-proBNP concentration and change in echocardiographic measurements LVESVi, LAVi, and LVEF from baseline to month 6; change from baseline in concentration of NT-proBNP and change in LAVi, LVESVi and LVEF by selected groups of interest at month 6, and other times	52 weeks

BNP, Brain natriuretic peptide; cGMP, Cyclic guanosine monophosphate; ECHO, Echocardiography; EROA, Effective regurgitant orifice area; HF, Heart failure; KCCQ, Kansas City Cardiomyopathy Questionnaire; LVEF, Left ventricular ejection fraction; LAVi, Left atrial volume index; LVEDVi, Left ventricular end diastolic volume index; LVESVi, Left ventricular end systolic volume index; NYHA, New York heart association; NT-proBNP, N-terminal pro-B-type natriuretic peptide.

**Table 3:** Results of clinical trials with sacubitril/valsartan.

Trial	Main results	Extended results	Ref.
PARAMOUNT	NT-proBNP↓ Reversal of left atrial remodeling Improvement in NYHA class		(37)
		hs-TnT↓	(38)
		IGFBP7↓	(39)
PARADIGM-HF	The risks of death and of hospitalization for HF↓ The number of patients who went to the emergency department due to the need for intensive drug treatment of HF or deterioration of the disease↓. The risk of HF deterioration↓. Biomarkers of myocardial wall stress and injury (NT-proBNP and hs-TnT)↓		(40, 41)
		Cardiovascular death and HF hospitalization throughout the LVEF spectrum↓	(42)
		Sudden cardiac deaths and deaths from worsening HF↓ More beneficial than enalapril across the spectrum of ages with a favorable benefit-risk profile in all age groups.	(43, 44)
		Change scores in KCCQ clinical summary scores and KCCQ overall summary scores are better than enalapril. It significantly improves nearly all KCCQ physical and social activities compared with enalapril, with the largest responses in household chores and sexual relationships.	(45, 46)
		The risk of hyperkalemia↓	(47)
		The possibility of reducing NT-proBNP to ≤1,000 pg/ml with sacubitril/valsartan treatment is nearly twice as high as that with enalapril.	(48)
		sST2 and biomarkers (TIMP-1, MMP-9, PINP, PIIINP, and others) that reflect the stability of ECM↓	(49, 50)
PARAGON-HF	Primary outcome events↓ No significant difference in the risk of death from cardiovascular causes and in the rate of hospitalizations. The change in the NYHA class and the occurrence of declining renal function favors sacubitril/valsartan over valsartan. Sacubitril/valsartan is associated with a higher incidence of hypotension and angioedema but a lower incidence of elevated serum creatinine or potassium levels.		(51)
		The incidence of primary outcomes↓	(52)
		The risk of heart failure hospitalization in women than in men↓	(53)
		The risk of renal events and decline in the eGFR↓	(54)
		Triglycerides↓	(55)
		Biomarkers reflecting extracellular matrix homeostasis↓	(56)
TRANSITION	Approximately half of HFrEF patients achieved the recommended target dose of sacubitril/valsartan within 10 weeks after an ADHF event, and 86% or more tolerated any dose of sacubitril/valsartan for over 2 weeks. Adverse events and permanent treatment discontinuations were low.		(57)
		The target dose is achieved in more *de novo* HFrEF patients than prior HFrEF patients, and fewer had SAEs and permanent treatment discontinuation. *De novo* patients show faster and greater decreases in NT-proBNP and hs-TnT, and lower rates of HF and all-cause rehospitalization vs. prior HFrEF.	(58)
PRIME	MR among patients with secondary functional MR↓ The number of patients with symptomatic hypotension, angioedema, a serum creatinine level of ≥2.5 mg/dl, and hyperkalemia did not differ between the treatment groups.		(59)
PIONEER-HF	NT-proBNP concentration among patients with HFrEF who were hospitalized for ADHF↓ Rates of worsening renal function, hyperkalemia, symptomatic hypotension, and angioedema did not significantly differ.		(60)
		hs-TnT and sST2 in patients with ADHF↓	(61)
		Patients that began taking sacubitril/valsartan in the hospital over the entire 12 weeks of follow-up had a lower hazard for the composite outcome compared with patients that initiated enalapril in the hospital followed by sacubitril/valsartan initiation 8 weeks later.	(62)
EVALUATE-HF	aortic Zc↓ Zc in women with LVEF ≥ 40%↓		(63)
		The LVEDVI, LVESVI, LAVI and E/e’ ratio↓ NT-proBNP, sST2, and hs-TnT↓ The urinary cyclic guanosine monophosphate/urinary creatinine ratio↑	(64)
PROVE-HF	The decrease of NT-proBNP concentration correlates with the improvement of cardiac volume and functional markers. Improvement in Hs-TnT levels, ANP levels, and KCCQ-23 scores.		(65–67)

ADHF, acute decompensation event; ECM, extracellular matrix; eGFR, estimated glomerular filtration rate; HF, Heart failure; HFrEF, HF with reduced ejection fraction; hs-TnT, high-sensitivity troponin T; KCCQ, Kansas City Cardiomyopathy Questionnaire; LAVI, left atrial volume index; LVEDVI, left ventricular end-diastolic volume index; LVEF, Left ventricular ejection fraction; LVESVI, left ventricular end-systolic volume index; MMP-9, matrix metalloproteinase-9; MR, mitral regurgitation; NT-proBNP, N-terminal pro-B-type natriuretic peptide; NYHA, New York Heart Association; PIIINP, N-terminal propeptide of collagen III; PINP, N-terminal propeptide of collagen I; sST2, soluble suppression of tumorigenicity 2; TIMP-1, tissue inhibitor of matrix metalloproteinase-1.

Jhund et al. found by testing high-sensitivity troponin T (hs-TnT) in patients from the PARAMOUNT trial that most had hs-TnT concentrations above the threshold for diagnosing myocardial injury (38). Higher hs-TnT concentrations were associated with increased age, elevated NT-proBNP levels, and lower estimated glomerular filtration rate (eGFR), suggesting a poorer prognosis for HFpEF. The reduction in hs-TnT was greater after 36 weeks of sacubitril/valsartan treatment than after valsartan treatment. Sacubitril/valsartan may attenuate myocardial injury in patients with HFpEF by enhancing the NPs system. Insulin-like growth factor-binding protein-7 (IGFBP7) is a senescence-associated protein that inhibits cell proliferation by arresting the cell cycle. Its elevated circulating concentrations is associated with cardiac hypertrophy, abnormal ventricular filling dynamics, and poor prognosis in patients with HFpEF (68). Januzzi et al. found that IGFBP7 concentrations in patients with HFpEF predicted the presence of left atrial dilation based in 228 participants from the PARAMOUNT trial (39). Interestingly, sacubitril/valsartan treatment leads to a decrease in IGFBP7 concentrations that is not observed in valsartan-treated patients. This decrease in IGFBP7 may be a direct effect of NEPI on age-related pathways or an effect of LBQ657 on the underlying pathophysiological processes of the disease; this needs further investigation (Table 3).

### 3.2. PARADIGM-HF

PARADIGM-HF (NCT01035255) is a multicenter, randomized, double-blind, parallel-group, active-controlled phase III clinical trial that compares the long-term efficacy and safety of enalapril and sacubitril/valsartan in patients with HFrEF (69). The trial was stopped early after a median follow-up of 27 months produced overwhelming benefits of sacubitril/valsartan. The 2014 results noted a significant advantage of sacubitril/valsartan over enalapril in reducing cardiovascular mortality, risk of HF hospitalization, HF symptoms, and physical limitations (40). Sacubitril/valsartan-treated patients require significantly fewer visits to the emergency department for intensive drug therapy for HF or for disease progression and have a reduced risk of worsening HF. The use of sacubitril/valsartan significantly reduces the rate of HF hospitalization while lowering NT-proBNP and hs-TnT levels during the first 30 days after randomization; this stabilizing effect on the course of HF may have an important impact on patient's quality of life (41).

Solomon et al. correlated the results of the PARADIGM-HF study with LVEF to assess the effectiveness of Sacubitril/Valsartan across the LVEF spectrum. The LVEF is a significant and independent predictor of all clinical outcomes in patients with HFrEF enrolled in the trial. Each 5% reduction in LVEF is associated with a 9% increase in the risk of cardiovascular death or hospitalization for HF. Sacubitril/valsartan effectively reduces cardiovascular death and hospitalization for HF across the LVEF spectrum (42). A study of the mode of death of deceased patients reveals that sacubitril/valsartan reduces cardiovascular mortality by 20% by decreasing the incidence of sudden death and progressive HF; however, it has no significant effect on the incidence of non-cardiovascular death (43). Sacubitril/valsartan reduces mortality in HFrEF, primarily by reducing cardiovascular death due to worsening HF. It has a significant benefit compared with enalapril across the age range of PARADIGM-HF, despite increasing mortality and HF hospitalization rates with patient age (44).

HF negatively affects health-related quality of life (HRQL) in the physical, psychological, and social domains. HRQL predicts the risk of future morbidity and mortality and is a key target for the treatment of patients with chronic HF (70). The Kansas City Cardiomyopathy Questionnaire (KCCQ) is an HF-specific HRQL test validated in patients with HFrEF (71). The KCCQ clinical summary score (KCCQ-CS) and KCCQ overall summary score (KCCQ-OS) are better in patients treated with sacubitril/valsartan than in those treated with enalapril and this trend persisted over the 8 month follow-up period (45). Furthermore, sacubitril/valsartan significantly improves physical and social activities in almost all HFrEF patients, especially in the areas of housework and sexual relationships (46).

The PARADIGM-HF trial also found that the risk of severe hyperkalemia is significantly reduced when mineralocorticoid receptor antagonists (MRA) are combined with sacubitril/valsartan, but not enalapril (47). Preferential use of sacubitril/valsartan in eligible patients with HFrEF improves the safety of MRA and allows patients to benefit with less risk.

Decreased NPs levels are associated with decreased morbidity and mortality, while increased NPs levels suggest a poor prognosis (48). Patients whose NT-proBNP level decreased to below 1,000 pg/ml have a 59% lower risk of the primary endpoint event than those who did not. The median NT-proBNP values are significantly lower in patients treated with sacubitril/valsartan than in those treated with enalapril after a month of randomization. Furthermore, the sacubitril/valsartan treatment is nearly twice as likely to reduce NT-proBNP levels to ≤1,000 pg/ml compared with enalapril. Elevated levels of soluble suppression of tumorigenicity 2 (sST2) correlate with the severity of adverse cardiac remodeling and fibrosis, and represent a potential prognostic biomarker for HFrEF. The sST2 levels decreased to a greater extent over time in patients from the sacubitril/valsartan treatment group than in those in the enalapril group in the PARADIGM-HF trial (49). In addition, sacubitril/valsartan treatment improves biomarkers reflecting extracellular matrix homeostasis (including TIMP-1, MMP-9, PINP, and PIIINP), reduces collagen synthesis and processing *in vivo*, and inhibits myocardial fibrosis (50).

### 3.3. PARAGON-HF

PARAGON-HF (NCT01920711) is a multicenter, randomized, double-blind, parallel-group, active-controlled, phase III clinical trial. The efficacy and safety of sacubitril/valsartan was compared to valsartan through assessing the reduction in cardiovascular mortality and overall HF hospitalization rates in patients with HFpEF. PARAGON-HF is the largest outcome trial conducted to date in patients with HFpEF (72), with more stringent inclusion criteria than those of previous trials.

The results of the study published in 2019 suggested that Sacubitril/valsartan has fewer primary outcome events than valsartan (51). The sacubitril/valsartan group has a lower (but not statistically significant) rate of HF hospitalization, along with no significant difference in the risk of cardiovascular mortality. Sacubitril/valsartan did not significantly reduce the overall hospitalization rate for HF and mortality from cardiovascular causes in patients with HFpEF. Sacubitril/valsartan is superior to valsartan in improving the NYHA classification and reducing the decline in renal function in four exploratory secondary outcomes. In terms of safety, Sacubitril/Valsartan is associated with a higher incidence of hypotension and vasogenic edema and a lower incidence of elevated creatinine or potassium ions. Pooled analysis of data from PARADIGM-HF (LVEF ≤ 40%; *n* = 8,399) and PARAGON-HF (LVEF ≥45%; *n* = 4,796) and grouped studies based on LVEF show a significantly lower incidence of primary outcome in patients treated with sacubitril/valsartan than in those treated with ACEI/ARB (52). Hospitalization for HF, cardiovascular death, and all-causes of mortality significantly decrease as LVEF increases, with the greatest decrease in cardiovascular mortality and a smaller decrease in hospitalization for HF. In particular the effect on outcomes continue across the spectrum of EF until 50% for men and 55% for women. Infact, the PARAGON-HF trial results suggest that sex alters the effect of sacubitril/valsartan vs. valsartan on the primary outcome event; the drug worked better for women than for men. Also, sacubitril/valsartan is more likely to reduce the risk of hospitalization for HF in female patients than valsartan (53).

The therapeutic benefit of sacubitril/valsartan in patients with HFpEF is amplified in the high-risk window following hospitalization compared with valsartan, and it potentially reduces the additional risk associated with the high-risk period; however, prospective trials are needed to validate this finding. Over time, sacubitril/valsartan does not significantly reduce the burden of HF signs and symptoms; however, it reduces the incidence of exertional dyspnea compared with valsartan and significantly reduces the incidence of renal composite outcomes and the overall rate of decline in eGFR (54).

Sacubitril/valsartan significantly reduces triglyceride levels by almost three-fold in patients with elevated triglyceride levels at baseline (55). In addition, sacubitril/valsartan reduces sST2, TIMP-1, and PIIINP levels, and increases CITP levels, inhibits profibrotic signaling, and this may be an important mechanism for the benefit observed on outcomes (56).

### 3.4. TRANSITION

Although the PARADIGM-HF trial shows that sacubitril/valsartan is superior to ACEI in reducing cardiovascular morbidity and mortality in HFrEF, only 2.3% of HFrEF in patients in the United States who were able to receive ARNI therapy (nearly 70% of HFrEF patients) were treated with sacubitril/valsartan at discharge (73). This may be owing to the lack of sufficient evidence on the benefit and safety of in-hospital sacubitril/valsartan initiation in the HFrEF patient population. The TRANSITION test is a good complement to the PARADIGM-HF test (57). TRANSITION (NCT02661217) is a multicenter, randomized, open-label, parallel-group phase IV clinical trial comparing sacubitril/valsartan treatment in patients with HFrEF admitted for an acute decompensation event (ADHF) administered at different times before and after hospital discharge (74).

Approximately half of the patients with HFrEF achieved the recommended target dose of sacubitril/valsartan within 10 weeks after ADHF; over 86% tolerated any dose of sacubitril/valsartan for over 2 weeks, and the percentage of patients who permanently discontinued sacubitril/valsartan owing to adverse events was low (57). A higher proportion of patients with new-onset HFrEF tolerated the target dose at week 10, and fewer experienced serious adverse events leading to permanent therapy discontinuation compared with patients with previously diagnosed HFrEF (58). Early intervention with sacubitril/valsartan may have greater benefits in delaying disease progression in patients with new-onset HFrEF. In addition, new-onset patients have a faster and greater decline in NT-proBNP and hs-TnT levels, and lower all-cause rehospitalization rates. In-hospital administration of sacubitril/valsartan results in a rapid and significant reduction in NT-proBNP levels: NT-proBNP is reduced by 28% at discharge among patients treated with sacubitril/valsartan in-hospital, and 46% of patients have a reduction in NT-proBNP from baseline levels to ≤1,000 pg/ml.

### 3.5. PRIME

PRIME (NCT02687932) is a prospective, multicenter, double-blind, randomized, active-controlled trial. This study investigated the effects of sacubitril/valsartan and valsartan in patients with functional mitral regurgitation (MR) secondary to left ventricular dysfunction by measuring the effective regurgitant orifice area (EROA), regurgitant flow, and cardiac function parameters. Sacubitril/valsartan reduces MR more than valsartan in patients with secondary functional MR. An intention-to-treat analysis of 117 patients shows that the sacubitril/valsartan and valsartan groups have significant differences in EROA (30% and 9% decrease, respectively), and in return flow at the study endpoint (33% and 12% decrease, respectively). The left ventricular end-systolic and end-diastolic volumes were significantly reduced in the sacubitril/valsartan group. In addition, there is no difference in the proportion of patients with adverse effects, such as symptomatic hypotension, angioedema, elevated serum creatinine levels, and hyperkalemia between the two treatment groups during the follow-up period (59). These findings support the positive effect on LV remodeling and mitral regurgitation in HFrEF patients.

### 3.6. PIONEER-HF

PIONEER-HF (NCT02554890) is a multicenter, randomized, double-blind, active-controlled trial in patients with ADHF with reduced ejection fraction compared with in-hospital initiation of sacubitril/valsartan therapy or enalapril therapy. PIONEER-HF is the first clinical trial to investigate the effectiveness and safety of sacubitril/valsartan treatment in patients with ADHF.

In-hospital initiation of sacubitril/valsartan treatment results in a greater reduction in NT-proBNP concentrations than enalapril treatment in patients with HFrEF hospitalized for ADHF. The decrease in NT-proBNP concentration is more pronounced in the sacubitril/valsartan group than in the enalapril group at weeks 4 and 8, and the variability in the magnitude of this decrease was particularly significant at week 1. Safety analysis shows no significant difference in the incidence of worsening renal function, hyperkalemia, and symptomatic hypotension between the sacubitril/valsartan and enalapril groups, and the rate of permanent discontinuation of the trial drug owing to adverse events is not significantly different between the two treatment groups (60). The efficacy and safety are not affected by previous history of HF, whether ACEI/ARB treatment is administered, or whether the target dose is achieved (75–77).

Post hoc analysis shows that sacubitril/valsartan significantly reduces hs-TnT and sST2 in patients with ADHF and parallels the decrease in NT-proBNP (61). This suggests that sacubitril/valsartan may reduce myocardial injury and improve hemodynamics at an early stage. In the continuing 4-week study, patients who started sacubitril/valsartan in the hospital have a lower incidence of HF rehospitalization or cardiovascular death than patients who started sacubitril/valsartan after 8 weeks of taking enalapril in the hospital. It is worth noting that change from enalapril to sacubitril/valsartan in the patient after 8 weeks results in a further 37% reduction in NT-proBNP levels (62). This indicates that delayed sacubitril/valsartan treatment still produces a large benefit to patients.

### 3.7. EVALUATE-HF

EVALUATE-HF (NCT02874794) is a randomized, double-blind, multicenter clinical trial investigating the effects of sacubitril/valsartan on aortic sclerosis and cardiac remodeling in patients with HFrEF compared with enalapril.

Sacubitril/valsartan did not significantly improve central aortic sclerosis in patients with HFrEF compared to enalapril. The primary endpoint of aortic characteristic impedance (Zc) decreases in the sacubitril/valsartan group and slightly increases in the enalapril group from baseline to 12 weeks, however the differences between the treatments are not statistically significant. Meanwhile, there was a significantly greater reduction in brachial artery systolic pressure in the sacubitril/valsartan group than in the enalapril group. Treatment with sacubitril/valsartan is associated with a more pronounced reduction in Zc in female patients from the subgroup of patients with baseline LVEF ≥40%. However, no sex difference is observed in the group of patients with LVEF <40% (63).

At the secondary endpoint, patients in the sacubitril/valsartan group showed greater decreases in left ventricular end-diastolic volume index (LVEDVI), left ventricular end-systolic volume index (LVESVI), left atrial volume index (LAVI), and the *E*/*e*, ratio from the baseline to week 12 compared with the enalapril group. However, LVEF did not significantly differ between groups during this period. The sacubitril/valsartan group showed greater reductions in NT-proBNP, sST2, and hs-cTnT levels at 12 weeks, while the urinary cyclic guanosine phosphate/creatinine ratio increases at 12 weeks. Post hoc analyses showed that changes in NT-proBNP significantly associate with changes in left ventricular volumes, and the improvement in the total KCCQ score is significantly higher in the sacubitril/valsartan group than in the enalapril group. A higher proportion of patients in the sacubitril/valsartan group show an improvement in the total KCCQ score of ≥5 points, and changes in the quality of life are associated with improvements in NT-proBNP. In terms of safety, incidence of hypotension, hyperkalemia, and deterioration of renal function is similar in both groups (64).

### 3.8. PROVE-HF

In previous studies, Sacubitril/valsartan treatment is strongly associated with a decrease in NT-proBNP levels in patients with HF. However, it is unclear whether the beneficial effect of sacubitril/valsartan in patients with HF is associated with the reversal of myocardial remodeling. PROVE-HF (NCT02887183) is a 12-month prospective, single-arm, open-label trial investigating the correlation between changes in NT-proBNP and long-term changes in cardiac remodeling measures after the initiation of sacubitril/valsartan in patients with HFrEF.

These results suggest that the reduction in NT-proBNP concentration correlates with improvements in cardiac volume and functional markers at 12 months. The change in NT-proBNP concentration significantly correlates with LVEF, LVEDVI, LVESVI, LAVI, and *E*/*e*, from baseline to month 12. In addition, blood hs-cTnT and ANP levels and KCCQ-23 scores significantly improve after 12 months of sacubitril/valsartan treatment, and these improvements are closely associated with the reversal of myocardial remodeling (65–67). The cardioprotective effect of sacubitril/valsartan in HFrEF patients may be associated with the reversal of cardiac remodeling (78). The improvement in NT-proBNP levels and myocardial remodeling by sacubitril/valsartan did not significantly differ between patients of different ethnicities, although greater mean gains are seen in black patients in the first half of the trial and in white patients in the second half (79, 80). However, the explanation of this phenomena requires further.

### 3.9. Other clinical trials

A prospective single-center study of 108 patients with symptoms of HF shows that patients with HFrEF and severe right ventricular dysfunction have a worse prognosis than patients with mild right ventricular dysfunction after treatment with sacubitril/valsartan, although both groups show significant improvements in left and right ventricular function (81).

A multicenter study of 727 patients with HFrEF suggests that sacubitril/valsartan improves the hemodynamic status of patients. The magnitude of hemodynamic improvement reduced composite risk of all-cause mortality and re-hospitalization for HF during follow-up is associated with the dose of sacubitril/valsartan. The relationship between the maximum dose of sacubitril/valsartan tolerated by patients and clinical efficacy warrants further exploration (82).

The LIFE trial evaluated the tolerability, safety, and efficacy of sacubitril/valsartan in patients with advanced HFrEF (patients with NYHA class IV and LVEF ≤35%) vs. valsartan alone. Sacubitril/valsartan did not improve the clinical composite endpoints of days to survival, days to discharge, and absence of HF events compared with valsartan (83).

The TAROT-HF study found that patients with non-ischemic cardiomyopathy (NICM) generally show a greater improvement in LVEF than patients with ischemic cardiomyopathy (ICM) after sacubitril/valsartan treatment (84).

## 4. Disease application of sacubitril/valsartan

Sacubitril/valsartan may be an effective treatment for HF and is a potential therapeutic agent for other diseases (Tables 4.1, 4.2).

**Table 4.1:** Clinical trial of sacubitril/valsartan in other diseases.

Disease	Research object/Trial	Effect	Pathophysiological mechanisms	Ref.
Hypertension	Asian patients with hypertension	24 h, daytime, and nighttime ambulatory systolic BP, diastolic BP, and pulse pressure↓	–	(85)
	Elderly with systolic hypertension	Central aortic systolic and pulse pressure, mean 24 h ambulatory brachial systolic blood pressure, and mean seated pulse pressure↓	–	(86, 87)
	Asian patients with refractory hypertension	Office and ambulatory BP (especially nighttime ambulatory BP)↓	–	(88)
	Hemodialysis patients with resistant hypertension	Controlled resistant hypertension and improved cardiac structure and function	–	(89)
	Salt-sensitive hypertension patients	Natriuresis and diuresis↑, NT-proBNP↓	–	(90)
	HFpEF patients with hypertension	More significant blood pressure reduction than patients receiving valsartan alone	–	(37)
	Patients with resistant hypertension and MRA-resistant hypertension	Blood pressure is reduced more than patients receiving valsartan	–	(91)
Arrhythmias	HF-patients equipped with an ICD or CRT	VT/VF, appropriate therapy, NsVT, hourly PVC-burden↓	Cardiac reverse remodeling	(92)
	Chronic HF patients with LVEF ≤40%	QTc interval, QRS duration and mechanical dispersion index as assessed by LV GLS↓	–	(93)
	HFrEF patients with an ICD and remote monitoring	Compared to ACEI, arrhythmias, and appropriate ICD shocks↓	KCNN2 and CaMKII-p↓ ERG, KCNE1, and KCNE2 expression↑	(94–96)
	Patients with persistent AF who received RFCA	AF recurrence after catheter ablation↓		(97)
	HF patients in six RCTs	Similar to either ACEI or ARB in preventing the occurrence of AF for HF	–	(98)
PPH	HFpEF patients with pulmonary hypertension	Pulmonary artery pressure and mean pulmonary capillary wedge pressure↓	–	(99)
DM	PROVE-HF	Affects natriuretic peptide levels, reverses cardiac remodeling, and health status		(79)
	PARADIGM-HF	Occurrence of the primary composite outcome compared with enalapril↓	–	(100)
	PARADIGM-HF	Long-term HbA1c↓, the patients that started using insulin and oral antihyperglycemic therapy↓	–	(101)
	PARADIGM-HF/PARAGON-HF	Decline of eGFR and clinically relevant kidney events↓	–	(102, 103)
Renal insufficiency	Patients with HFrEF	Rate of decrease in the eGFR↓	–	(104)
	Patients with HF and CKD	eGFR↑	–	(105)
	PARAGON-HF	Lower decline in renal function	–	(54)

ACEI, Angiotensin-converting enzyme inhibitor; AF, Atrial fibrillation; BP, Blood pressure; CaMKII-p, Phosphorylated Ca2+/calmodulin-dependent protein kinase II; CRT, Cardiac resynchronization therapy; eGFR, Evaluate glomerular filtration rate; ERG, ETS-related gene; HbA1c, Hemoglobin A1c; HF, Heart failure; HFpEF, Heart failure with preserved ejection fraction; HFrEF, Heart failure with reduced ejection fraction; ICD, Implantable cardioverter defibrillator; KCNN2, Potassium calcium-activated channel subfamily N member 2; LV GLS, Left ventricular global longitudinal strain; LVEF, Left ventricular ejection fraction; MRA, Mineralocorticoid receptor antagonist; NT-proBNP, N-terminal pro-B-type natriuretic peptide; PVC, Premature ventricular contraction; RCTs, Randomized controlled trials; RFCA, Radio-frequency catheter ablation; VT/VF, Ventricular tachycardia/fibrillation.

**Table 4.2:** Experimental studies of sacubitril/valsartan in other diseases.

Disease	Research object	Effect	Pathophysiological mechanisms	Ref.
Arrhythmias	RAP induced rabbit model of AF	Atrial electrical remodeling and structure remodeling in AF↓	Calcineurin/NFAT pathway↓	(106)
	Rat AngII continuous subcutaneous stimulation	Extent of atrial fibrosis, proliferation, migration, and differentiation of atrial fibroblasts, and susceptibility to AF↓	p-Smad2/3, p-JNK, and p-p38MAPK pathways↓	(107)
PPH	PH induced by MCT and Sugen/hypoxia	Pulmonary pressures, vascular remodeling, and RV hypertrophy and fibrosis↓	–	(108)
	SU5416/hypoxia rat model	RV pressure↓	Pulmonary vascular wall thickness and plasma endothelin-1↓; lung levels of ANP, BNP, and cGMP↑	(109)
	RV pressure overload rats *via* banding the main pulmonary artery	RV maximum pressure↓, RV contractile and relaxation functions↑, RV afterload↓, and prevents RV-pulmonary artery uncoupling	Hypertrophy, collagen, and myofiber reorientation↓	(110)
	SU5416/hypoxia rat model	RV systolic pressure, hypertrophy, and dilatation↓	Pulmonary vascular remodeling↓	(111)
DM	Diabetic TGR(mREN2)27 rats	Proteinuria↓	–	(112)
	Male Zucker obese rats	Proteinuria, renal ultrastructure, and tubular injury↓	Mesangial expansion↓, improved podocyte and tubular mitochondrial ultrastructure	(113)
	Rats with T2DM	Complete reversal of the early decrease in thermal sensation and loss of sensory nerve fibers in the skin, thermal sensitivity, and intraepidermal nerve fiber density↑, and reversal of the vascular dysfunction demonstrated by vasodilation to acetylcholine	–	(114)
Renal insufficiency	Rats with CRS	BUN, creatinine, and ratios of urine protein to creatinine↓	Kidney weight, oxidative stress, and kidney fibrosis↓	(17)
	Mice with CRS	Sclerosis of the glomerulus, fibrosis of renal tubular, and NGAL↓	–	(115)
	Dogs with CRS	Signal prevention of ongoing kidney injury	KIM-1, NGAL, and plasma cystatin-C↓	(116)

Ang II, Angiotensin II; ANP, Atrial natriuretic peptide; ARB, Angiotensin-receptor blockers; BUN, Blood urea nitrogen; cGMP, Cyclic guanosine monophosphate; CKD, Chronic kidney disease; CRS, Cardiorenal syndrome; DM, Diabetes mellitus; KIM-1, kidney injury molecule-1; MCT, Monocrotaline; NFAT, Nuclear factor of activated T cells;NGAL, Neutrophil gelatinase-associated lipocalin; NsVT, Non-sustained ventricular tachycardia; PH, Pulmonary hypertension; p-JNK, Phospho-c-Jun N-terminal kinases; p-p38MAPK, Phosphorylated p38 mitogen-activated protein kinases; PPH, Primary pulmonary hypertension; RAP, Rapid atrial pacing; RV, Right ventricular; T2DM, Type 2 diabetes mellitus.

### 4.1. Hypertension

Sacubitril/valsartan significantly reduces the 24-hour daytime and nighttime dynamic systolic-, diastolic-, and pulse pressures. It is well tolerated with no reported cases of angioedema (85). Patients treated with sacubitril/valsartan have more significant decreases in central arterial systolic pressure, central aortic pulse pressure, mean 24-hour ambulatory brachial artery systolic pressure, and mean sitting diastolic pressure than those treated with ACEI/ARB (86, 87). Sacubitril/valsartan significantly reduces office and ambulatory blood pressure and substantially reduces nocturnal ambulatory blood pressure in 66 Asian patients with refractory hypertension (88). And sacubitril/valsartan controls refractory hypertension, significantly reduces mean seated systolic and diastolic blood pressure, and partially improves cardiac structure in a single-center prospective study of 360 hemodialysis patients with refractory hypertension (89). This more pronounced antihypertensive effect may be partly attributed to the better urinary sodium excretion in hypertensive patients treated with sacubitril/valsartan compared with ACEI/ARB (90); however, the exact mechanism is unclear.

The application of sacubitril/valsartan also appears to be of greater benefit than traditional ACEI/ARB in HF patients with hypertension. The PARAMOUNT study shows that patients with HFpEF treated with sacubitril/valsartan for 12 weeks have a more significant reduction in blood pressure than patients treated with valsartan and are better tolerated (37). Meanwhile, there is no significant difference in the incidence of hypotension between the two groups. Refractory hypertension affects 10%–20% of HFpEF patients each year; some investigators have examined treatment of HFpEF patients with refractory hypertension using sacubitril/valsartan is significantly more effective than treatment with valsartan in the PARAGON-HF trial (91).

The 2017 ACC/AHA Heart Failure Guidelines were updated to include ARNI as the preferred treatment for HF patients with hypertension in addition to conventional therapy since sacubitril/valsartan lowers blood pressure and improves the prognosis of HF patients with combined hypertension (117). However, the mechanism facilitating sacubitril/valsartan efficacy over traditional ACEI/ARB analogs requires further investigation.

### 4.2. Cardiac arrhythmia

Clinical studies show that replacing ACEI/ARB treatment with sacubitril/valsartan in patients with HFrEF reduces the degree of burden of ventricular tachycardia or ventricular fibrillation, results in fewer ICD interventions, and significantly reduces the number and duration of non-sustained ventricular tachycardia (92). Sacubitril/valsartan treatment decreases the number of patients with sustained ventricular tachycardia, and the number of episodes of sustained ventricular arrhythmia significantly decreases in symptomatic patients (93). Sacubitril/valsartan reduces persistent ventricular tachycardia/ventricular fibrillation, non-persistent ventricular tachycardia, and paroxysmal atrial tachycardia/atrial fibrillation(AF) episodes in patients with HFrEF implanted with an ICD (94). These effects may be closely related to the downregulation of cardiac KCNN2 and phosphorylated calmodulin-dependent protein kinase II expression, and the upregulation of ERG, KCNE1, and KCNE2 expression (95, 96).

Sacubitril/valsartan also has therapeutic effects on AF. In patients with persistent AF who underwent radiofrequency ablation the probability of AF is significantly lower in patients treated with sacubitril/valsartan after 12 months of intervention than in patients treated with valsartan for the same duration (97). However, sacubitril/valsartan treatment did not significantly differ from enalapril or valsartan in preventing the development of AF in patients with HF in a meta-analysis of a randomized, double-blind, active controlled trial involving 15,512 patients. Notably, the authors did not deny the therapeutic effect of sacubitril/valsartan on AF (98). Sacubitril/valsartan reduces the incidence of AF in rabbits with rapid atrial pacing by attenuating atrial electrical and structural remodeling (106) and may reduce AF susceptibility by inhibiting p-Smad2/3, p-JNK, and p-p38MAPK pathways against Ang II-induced atrial fibrosis (107).

Although there is still a lack of convincing studies to prove the advantages of sacubitril/valsartan over conventional antiarrhythmic drugs, the current study suggests that sacubitril/valsartan has a protective effect against ventricular arrhythmias and AF, which may provide new ideas for the clinical treatment of arrhythmias.

### 4.3. Primary pulmonary hypertension (PPH)

The severity of PPH symptoms and survival are closely related to right ventricular function and right heart failure is the leading cause of death in patients with PPH. However, there are no treatments that directly target the right ventricle. Long-term application of sacubitril/valsartan reduces right ventricular remodeling and fibrosis, improves right ventricular systolic and diastolic function, reduces right ventricular afterload, and significantly reduces pulmonary artery pressure (108–110). These effects appear to be secondary to pulmonary vascular changes, including reduced pulmonary vascular remodeling (111). Sacubitril/valsartan inhibits the proliferation of pulmonary artery smooth muscle cells in patients with idiopathic PPH *in vitro* (108).

We also came across a study on the therapeutic effect of sacubitril/valsartan on PPH in patients. In a retrospective case study of 18 patients with PPH and HFpEF, conversion of ACEI/ARB to sacubitril/valsartan resulted in a significant reduction in pulmonary artery pressure and mean pulmonary capillary wedge pressure on right heart catheterization compared to ACEI/ARB use, with 12 of these patients having improved NYHA functional class. Invasive measurements further showed a significant reduction in right atrial pressure at follow-up (99). Emily et al. included five patients with HF who were temporarily unable to receive heart transplantation due to concomitant severe PPH, four of whom received heart transplantation within 5–36 days after starting sacubitril/valsartan therapy without complications such as postoperative PPH, right heart failure, or postoperative hypotension requiring vasopressor support (118). These cases seem to suggest that sacubitril/valsartan could be a potential treatment for advanced HF patients with severe PPH awaiting cardiac transplantation. Whether sacubitril/valsartan has effects independent of improved cardiac function in patients with PPH still requires more studies to explore and discover.

### 4.4. Diabetes mellitus

A *post hoc* analysis of the PROVE-HF trial shows that 361 of 794 patients have type 2 diabetes mellitus (T2DM). Cross-sectional studies show that T2DM patients and non-T2DM show similar levels of improvement in LVEF and KCCQ-OS after 12 months of treatment. Similar changes are observed in echocardiographic measurements. Longitudinal analysis shows that the mean NT-proBNP significantly decreases in both groups, while KCCQ-OS scores and the total benefit is slightly greater in the non-T2DM group than in the T2DM group. These results suggest that patients with T2DM starting sacubitril/valsartan treatment gain at least similar benefits to health status and reversal of cardiac remodeling compared with patients without T2DM (79). It is noteworthy that HF patients with T2DM tend to have worse cardiac function and prognosis than non-T2DM HF patients using conventional therapy (100).

A secondary study of the PARADIGM-HF trial shows that sacubitril/valsartan results in a more significant benefit in HF patients with T2DM than ACEI analogs. Patients with T2DM have a higher risk of major composite outcomes such as HF hospitalization or cardiovascular death compared to patients without a history of T2DM, while sacubitril/valsartan reduces the incidence of major composite outcomes compared to enalapril and has higher KCCQ scores than the enalapril group after 8 months treatment (101). Patients using sacubitril/valsartan have a greater reduction in HbA1c than those using enalapril after 1 and 3 years of follow-up. The proportion of patients starting treatment using insulin and oral hypoglycemic agent is significantly lower in the sacubitril/valsartan group than in the enalapril group (119).

Sacubitril/valsartan also has a significant protective effect on renal function in patients with T2DM. Post hoc analysis of the PARADIGM-HF trial (102) and the PARAGON-HF trial (103) shows that patients with HFpEF and DM have a greater decrease in the eGFR than patients without DM. Sacubitril/valsartan treatment attenuates eGFR decline, reduces clinically relevant renal events, and improves prognosis in patients with DM and non-DM HFpEF compared with valsartan treatment. And sacubitril/valsartan is more effective than ARB alone in reducing urinary protein excretion in diabetic rats *in vivo* (112). These renoprotective effects may be associated with reduced thylakoid expansion and improved podocyte and tubular mitochondrial ultrastructures (113). Notably, these protective effects are not dependent on improvements in blood pressure, blood glucose level, or oxidative stress.

Sacubitril/valsartan also shows a more pronounced advantage over ACEI/ARB analogs in terms of its protective effects against diabetic neuropathy. Early intervention with sacubitril/valsartan completely reverses the early decline in thermal sensation and loss of sensory nerve fibers in the skin compared with valsartan in a diabetic rat model. Furthermore, it significantly improves or even reverses the diabetes-induced reduction in corneal nerve fiber length and sensitivity. Late intervention with sacubitril/valsartan significantly improves thermal sensitivity and partial intraepidermal nerve fiber density. In addition, sacubitril/valsartan reverses the impaired vascular responsiveness to acetylcholine and improves diabetes-induced vasculopathy (114).

These studies suggest that sacubitril/valsartan helps HF patients with diabetes to better control their blood glucose, fight, or delay the progression of complications to some extent, and improve their cardiac function compared with ACEI/ARB drugs. This is expected to provide greater benefit to patients with diabetes. However, it is notable that the protective effect of sacubitril/valsartan on diabetes-related complications is only demonstrated *in vivo*, and there are no reliable clinical trials showing that the drug retains these significant effects in patients. The mechanism of its improvement in related complications needs to be further investigated.

### 4.5. Renal insufficiency

Kevin et al. showed that sacubitril/valsartan is more effective in slowing the rate of decline in eGFR than enalapril, and positively impacts cardiovascular and renal outcomes in patients with HFrEF with and without CKD. These renal and cardiovascular benefits are observed despite the increased urinary albumin/creatinine ratio of sacubitril/valsartan compared with enalapril (104). A meta-analysis noted that sacubitril/valsartan significantly increases eGFR; however, there is no difference in the urinary albumin/creatinine ratio between sacubitril/valsartan and controls, including the irbesartan, valsartan, and enalapril groups (105).

A secondary study of the PARAGON-HF trial suggests that patients with HFpEF administered sacubitril/valsartan have >50% lower incidence of eGFR decline relative to the baseline and a lower rate of overall eGFR decline compared to valsartan (54). However, a different conclusion was reached in a randomized double-blind trial that included 414 participants with an eGFR of 20–60 ml/min/1.73 m^2^ that were randomly assigned to sacubitril/valsartan treatment vs. irbesartan treatment for over 12 months [UK HARP-III trial]. Sacubitril/valsartan has similar effects to irbesartan on renal function and proteinuria, although it lowers blood pressure and cardiac biomarkers in patients with CKD (120). The difference in results between the two trials may be due to the fact that participants in HARP-III were younger, predominantly male, had higher blood pressure, more severe renal insufficiency, higher proteinuria levels, and a very low self-reported prevalence of HF and diuretic use compared to PARAGON-HF participants (54).

The renoprotective effects of sacubitril/valsartan are further corroborated by *in vivo* experiments. Sacubitril/valsartan significantly reduces plasma creatinine and urea nitrogen levels and the urine protein/creatinine ratio in rats. This protective effect on renal function in rats may be owing to the reduction in renal mass, reversal of oxidative stress damage in the kidney, and improvement in renal fibrosis (17). Another study showed that sacubitril/valsartan improves glomerulosclerosis, tubular atrophy, and fibrosis in CRS mice to a greater extent than valsartan and reduces plasma NT-proBNP and urinary neutrophil gelatinase-related apolipoprotein, but does not significantly improve blood creatinine (115). Similar results are observed in the CRS canine model. Sacubitril/valsartan significantly reduces the levels of kidney injury markers (KIM-1, NGAL, and plasma cystatin C), but has no significant effect on blood creatinine and urea nitrogen (116). These results suggest that sacubitril/valsartan protects against ongoing kidney injury, but does not improve or worsen overall kidney function.

Studies have reached different conclusions on whether sacubitril/valsartan improves renal function in patients with HF and animal models. However, it is undeniable that it has a protective effect on the kidney, which may be due to the inhibition of renal fibrosis and protection against ongoing renal injury. Further studies are required to determine whether sacubitril/valsartan is more effective at combating the decline in renal function than traditional ACEI/ARB drugs.

## 5. Prospect & outlook

The latest 2022 ACC/AHA/HFSA guidelines for the management of heart failure state that sacubitril/valsartan is recommended as a Class IA treatment to reduce morbidity and mortality in patients with NYHA Class II-III HFrEF (8). Sacubitril/valsartan is recommended as a Class IB alternative to ACEI or ARB to further reduce morbidity and mortality in patients with NYHA class II-III HFrEF with chronic HF symptoms. However, clinical studies investigating the effect of sacubitril/valsartan on ventricular remodeling, myocardial fibrosis, and hemodynamic improvement are currently limited to patients with HFrEF. Further clinical trials are required to determine the involvement of sacubitril/valsartan in other CVD treatments including HFpEF, acute myocardial infarction, AF, and hypertension since they were performed using small sample sizes, insufficient study lengths, and incomplete observations. However, we are reasonably confident that sacubitril/valsartan will become a transgenerational drug in the field of CVD treatment in the near future.
